# *De Novo* Transcriptome of Safflower and the Identification of Putative Genes for Oleosin and the Biosynthesis of Flavonoids

**DOI:** 10.1371/journal.pone.0030987

**Published:** 2012-02-21

**Authors:** Haiyan Li, Yuanyuan Dong, Jing Yang, Xiuming Liu, Yanfang Wang, Na Yao, Lili Guan, Nan Wang, Jinyu Wu, Xiaokun Li

**Affiliations:** 1 Ministry of Education Engineering Research Center of Bioreactor and Pharmaceutical Development, Jilin Agricultural University, Changchun, Jilin, China; 2 College of Life Sciences, Jilin Agricultural University, Changchun, Jilin, China; 3 Institute of Genomic Medicine, Wenzhou Medical College, Wenzhou, China; University of Rome, Italy

## Abstract

Safflower (*Carthamus tinctorius* L.) is one of the most extensively used oil crops in the world. However, little is known about how its compounds are synthesized at the genetic level. In this study, Solexa-based deep sequencing on seed, leaf and petal of safflower produced a *de novo* transcriptome consisting of 153,769 unigenes. We annotated 82,916 of the unigenes with gene annotation and assigned functional terms and specific pathways to a subset of them. Metabolic pathway analysis revealed that 23 unigenes were predicted to be responsible for the biosynthesis of flavonoids and 8 were characterized as seed-specific oleosins. In addition, a large number of differentially expressed unigenes, for example, those annotated as participating in anthocyanin and chalcone synthesis, were predicted to be involved in flavonoid biosynthesis pathways. In conclusion, the *de novo* transcriptome investigation of the unique transcripts provided candidate gene resources for studying oleosin-coding genes and for investigating genes related to flavonoid biosynthesis and metabolism in safflower.

## Introduction

Safflower (*Carthamus tinctorius* L.), one of the most extensively used broadleaf plants in west Asia and China, is a source of conjugated linoleic acid (CLA) and an important herbal medicine with mild side effect [1]. Octadecadienoic acid, accounting for about 80% of safflower seed oil, can regulate cholesterol and is helpful in preventing cardiovascular disease [2], [3]. The major bioactive compound in safflower petals is flavonoid, which reportedly has many different pharmacological effects including preventing the occurrence of oxidation, inflammation, hypertension and cancer, and promoting blood circulation to dredge collaterals [4], [5]. In addition, safflower petals are commercially produced for use in the coloring and flavoring of foods, and for making dyes. Flavonoids include up to 5,000 kinds of secondary metabolites that exist in many plant species. Hydroxysafflor yellow A (HSYA), a chalcone glycoside, and safflor yellow B (SYB), a quinochalcone glycoside, are common ingredients of the flavonoids in safflower that are widely extracted and extensively used [6], [7]. Although flavonoid is biologically important, its synthesis pathways remain largely unknown. To date, no potential enzyme involved in catalyzing flavonoid biosynthesis has been discovered and annotated in safflower.

With the emergence of sequencing by synthesis (SBS) platforms, transcriptome characterization and expressed sequence tags (ESTs) analysis have become robust tools for identifying novel genes involved in specific biological pathways [8]. Next-generation sequencing provides not only large-scale identification of mRNA but also primary insight into functional genes involved in biological processes [9]. Solexa is a large-scale SBS platform that has been widely used in plant species in attempts to discover putative genes [10]. Safflower is a diploid plant whose genome has not yet been sequenced; this limits the understanding of molecular function and genomic structure in this plant. However, the *de novo* transcriptome could be useful and cost-effective for distinguishing transcripts, functional genes and for providing quantitative estimates of gene expression.

Considering the importance of safflower and the little knowledge that is available about its transcripts, we aimed to study the *de novo* transcriptome of the safflower leaf, petal and seed. We also aimed to identify the transcripts that were involved in the biosynthesis of the flavonoids that exist mainly in the safflower petal and partially in the leaf. As results, a total of 153,769 unigenes were generated by Illumina Solexa *de novo s*equencing technology; 39,390 of the unigenes from the seed were assembled into 68,889 scaffolds, 35,354 of the unigenes from the leaf were assembled into 51,702 scaffolds; and 60,628 unigenes from the petal were assembled into 100,650 scaffolds. Furthermore, 23 candidate unigenes from the petal cDNA library were characterized to be responsible for the biosynthesis of flavonoids. Additionally, 8 unigenes were putatively characterized as seed-specific oleosins. The data and results from this study will help drive novel functional gene discovery in researches into other plant species.

## Results

### Reads generation and assembly

Three safflower cDNA libraries from seed, leaf and petal were subjected to Solexa sequencing to explore their *de novo* transcriptomes. After removing the low quality reads and trimming off the adapter sequences, we obtained 60,269,546, 57,201,466 and 56,960,100 clean reads for the seed, leaf and petal transcriptomes respectively. The average length of the reads was 75 bp. An overview of the sequencing and assembly is given in table 1. All the short reads were deposited in the National Center for Biotechnology Information (NCBI) and can be accessed in the Short Read Archive (SRA) (accession number SRA047279.2).

**Table 1:** The summary of sequencing and assembling results.

		Sample	HQ sequences(n)	total bases(bp)	average length(bp)	gap sequences(n)	gap distribution (N/size)
**reads**	**length = 75**	seed	60,269,546	4,520,215,950	75	-	-
		leaf	57,201,466	4,290,109,950	75	-	-
		petal	56,960,100	4,272,007,500	75	-	-
**contig**	**length>100**	seed	152,639	38,007,111	249	-	-
		leaf	117,195	35,978,865	307	-	-
		petal	246,580	57,946,300	235	-	-
**scaffold**	**length>200**	seed	68,889	34,375,611	499	25,206	0.01–0.554
		leaf	51,702	33,761,406	653	18,205	0.01–0.565
		petal	100,650	53,143,200	528	44,659	0.01–0.627
**unigene**	**length>300**	seed	39,190	27,119,480	692	17,586	0.01–0.485
		leaf	35,354	29,697,360	840	11,829	0.01–0.395
		petal	60,628	43,227,764	713	30,361	0.01–0.464

**Figure 1:**
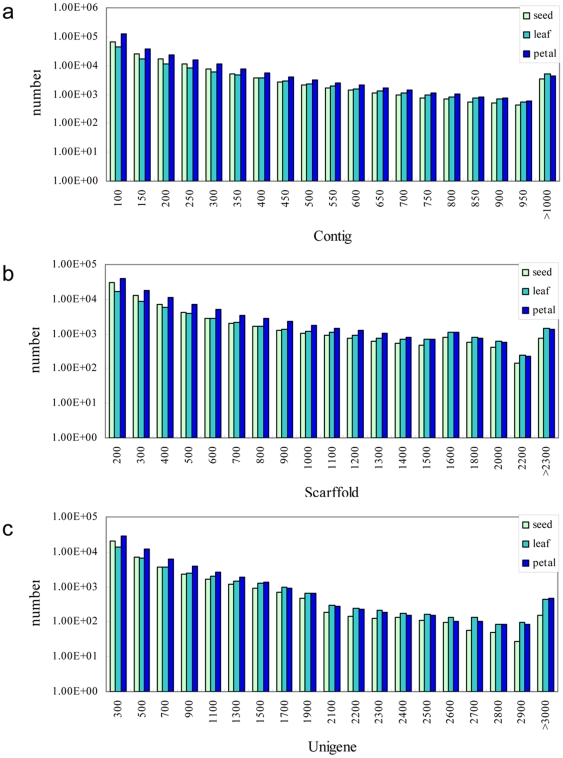
Overview of the sequencing and assembly of the safflower transcriptome. **A**. Overview of contig assembly. **B**. Overview of scaffold assembly. **C**. Overview of unigene assembly.

Because no reference genome sequence was available for safflower, all the clean reads (174,431,112) were assembled using SOAPdenovo [11]. 516,414 contigs (length >100 bp) were obtained ranging from 101 to 7,600 bp in length; the average size exceeded 235 bp. Assembled reads of the seed, leaf and petal accounted for 29.56% (average length 249 bp), 22.69% (307 bp) and 47.75% (235 bp) of the corresponding clean reads, respectively. The size distribution of the contigs is shown in figure 1A.

A total of 221,241 scaffolds were further assembled using the pair-end information of the assembled contigs. The total numbers of scaffold >200 bp long generated in the seed, leaf and petal libraries was 68,889 (average length of 499 bp), 51,702 (653 bp) and 100,650 (528 bp), respectively. Because the scaffolds were obtained from contigs using pair-end alignment, it was easier to estimate their length; however, the shortage was that part of scaffolds contained gaps. The percentage of gaps within scaffolds sequences ranged from min 1% to max 62.7%. In the seed, leaf and petal libraries, 25,206, 18,205 and 44,659 sequences were derived from high quality assembled scaffolds respectively. The size distribution of the scaffolds is shown in figure 1B.

The scaffolds were further assembled into unigenes with pair-end annotation. In total, 153,769 unigenes were generated from the seed, leaf and petal libraries of which 39,190 (average length of 692 bp), 35,154 (840 bp) and 60,628 (713 bp) were more than 300 bp long, respectively. The gapped sequences ranged from min 1% to max 62.7%, including 17,586, 11,829 and 30,361 sequences in seed, leaf and petal, respectively. The size distribution of the unigenes is shown in figure 1C.

### Functional annotation

A set of 153,769 unigenes were annotated using BLASTX and a variety of protein databases taking into account the identity between the unigene sequence and the sequence in the database (E value<0.00001). A total of 82,916 (53.92%), 52,842 (34.36%), 28,538 (18.56%) unigenes were aligned against the Nr, SWISS-PROT, and COG databases, respectively. The unmatched unigenes may represent tissue-specific novel genes.

**Figure 2:**
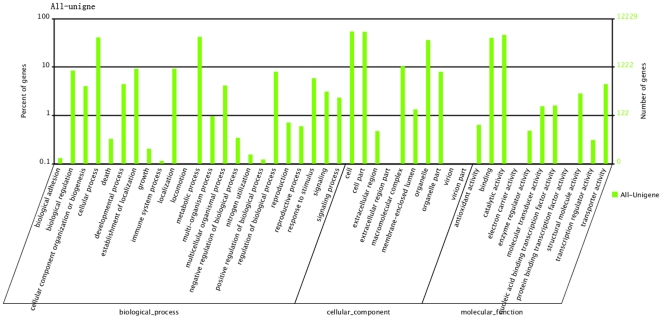
GO categories assigned to the safflower unigenes.

We used the GO (gene ontology) functional annotations from these databases to assign molecular function, cellular component and biology process terms to the safflower unigenes (figure 2). As a result, some of the genes in the seed, leaf and petal libraries were annotated with GO terms and 12,230 unigenes were assigned at least one GO functional category. The most abundant unigene GO terms were related to cellular components or to the molecular function of catalysis, indicating that these genes were enriched in the safflower transcriptome libraries. In addition, of the transcripts annotated with biological process terms, the most common were response to stimulus and response to stress.

### Candidate genes from the seed transcriptome involved in oleosin biosynthesis

In sunflower (*Helianthus annuus* L.), oleosin was reported to prevent the degradation of the seed oil body, particularly during seed desiccation [12], because it does not provide a binding site for the lipases that digest the oil body. A number of cDNAs encoding oleosins have been cloned from different plant seeds including *Arabidopsis thaliana*, sunflower (*H. annuus*), maize (*Zea mays*), rape (*Brassica napus*), and safflower (*C. tinctorius L*.) [13]. In the transcriptome of the safflower seed we identified eleven unigenes, unigene24748, unigene24871, unigene29297, unigene42440 and unigene76676, unigene76868, unigene80266, unigene83809, unigene83847, unigene120350 and unigene141701, that were annotated as candidate oleosins (table S1). These eleven unigenes may be involved in seed lipid storage and were selected for further analysis. The amino acid sequences of oleosins always have a central hydrophobic domain that is highly conserved between species. Therefore, it is likely that this domain is essential for oleosin function and it is thought to be inserted into the hydrophobic core of the oil body. Multiple sequence alignment of the translated amino acid sequences of these unigenes revealed that unigene80266, unigene83847 and unigene76868 had a hydrophobic domain in their sequences. These three unigenes were regarded as candidate oleosins that may be involved in the biosynthesis of octadecadienoic acid and they will be subjected to further research.

### Candidate genes from the petal transcriptome involved in the flavonoid biosynthesis pathway

Biological pathways, including metabolic pathways, signal transduction pathways, and genetic information processing pathways, were identified by KEGG pathway analysis of the unigenes. A total of 219 pathways were evaluated and some of the significant pathways that we found are listed in table S2. Significant pathways containing a large number of unigenes were pathways for photosynthesis, amino acid metabolism, histidine metabolism, and pathways for the biosynthesis of polyketide sugar unit, anthocyanin, betalain, carotenoid and indole, and flavone and flavonol.

**Figure 3:**
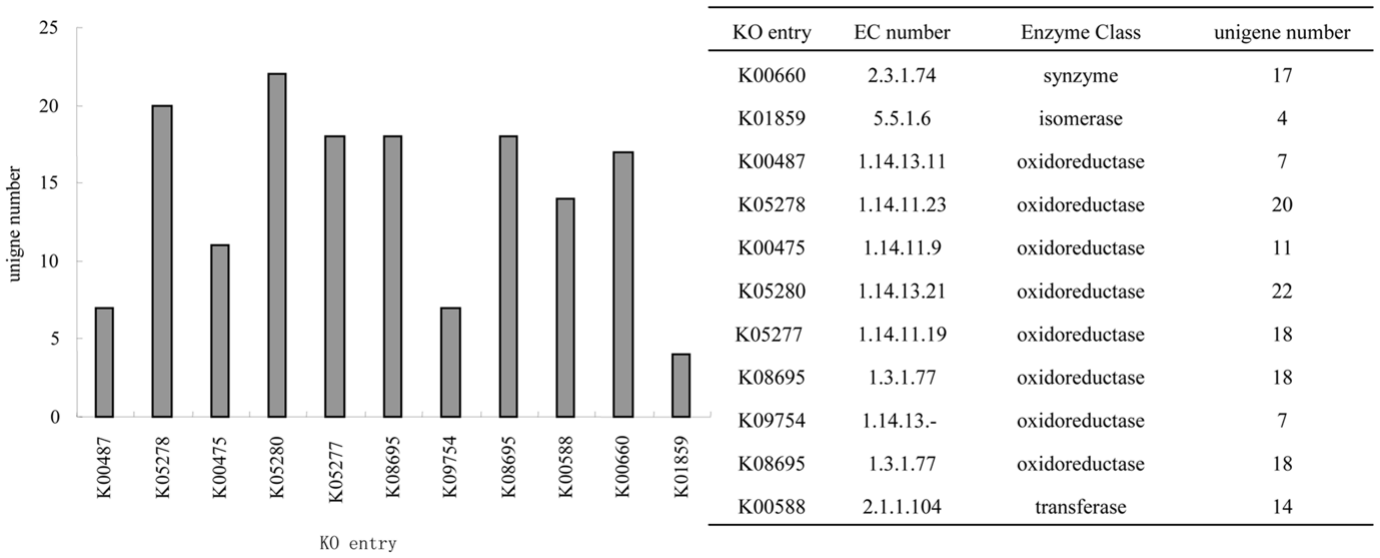
Unigenes predicted to be associated with flavonoid biosynthesis.

**Figure 4:**
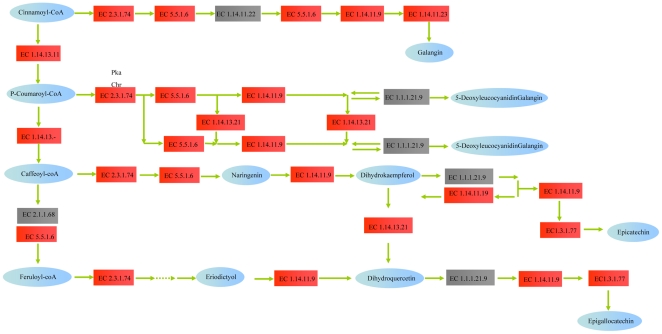
Unigenes predicted to be involved in the flavonoid biosynthesis pathway. Red indicates significantly increased expression; gray indicates genes that were not identified in the expression profile analysis; blue indicates unigenes predicted to be involved in the pathway.

Flavonoid is generally synthesized via the flavonoid biosynthesis pathway. 156 unigenes were annotated as encoding enzymes involved in flavonoid synthesis based on the KEGG pathway assignments (figure 3) and we investigated some of them. Chalcone synthase, an important enzyme involved in the flavonoid biosynthesis pathway, catalyzes the conversion of cinnamoyl-CoA to pinocembrin chalcone. It was reported that chalcone is a major ingredient of flavonol and that it plays an important role in flower development [14]. 17 unigene sequences (including unigene34109, unigene46610, and unigene53415 from the petal and leaf libraries were annotated as chalcone synthase. Chalcone isomerase is another important enzyme in the flavonoid biosynthesis pathway that catalyzes the conversion of pinocembrin chalcone to pinocembrin, a substrate of galangin synthesis. Altogether, we identified four unigenes as promising candidate chalcone isomerases. No genes encoding enzymes related to hydroxysafflor yellow were identified among the unigenes that we identified as candidate genes in the safflower flavonoid biosynthesis pathway. However, a number of unigenes related to enzyme families involved in flavonoid biosynthesis were identified, and these might prove to be potentially helpful for hydroxysafflor yellow enzyme discovery in the future (figure 4).

### Transcripts differentially expressed in the seed, leaf and petal libraries

To investigate the expression patterns of tissue-specific unigenes in the seed, leaf and petal libraries, the numbers of reads assembled from each library were evaluated separately for each unigene. We found that most of the unigenes involved in protein biosynthesis and biological regulation were highly expressed. These genes were mainly annotated as cellular development, for example, unigene19622 involved in protein synthesis was expressed almost equally and ubiquitously throughout the seed (637 reads), leaf (501 reads) and petal (588 reads). As expected, seed-specific unigenes were highly expressed only in the seed library; for example, unigene24748 annotated as oleosin was highly expressed (121,878 reads) in the seed and only slightly expressed in the leaf (639 reads) and the petal (107 reads); the expressions of unigene24871 and unigene29297 were similar. This expression pattern of the candidate oleosin genes is consistent with the earlier observations that oleosins are detected only in the seed of plants. The unigenes that were found to be highly expressed in all three libraries are likely to play important roles in growth and metabolism (e.g. unigene96376, unigene137965, and unigene14674). Other transcripts that were expressed differently in the three libraries (e.g. unigene64133, unigene82122, and unigene23361) may be involved in tissue-specific functions.

**Figure 5:**
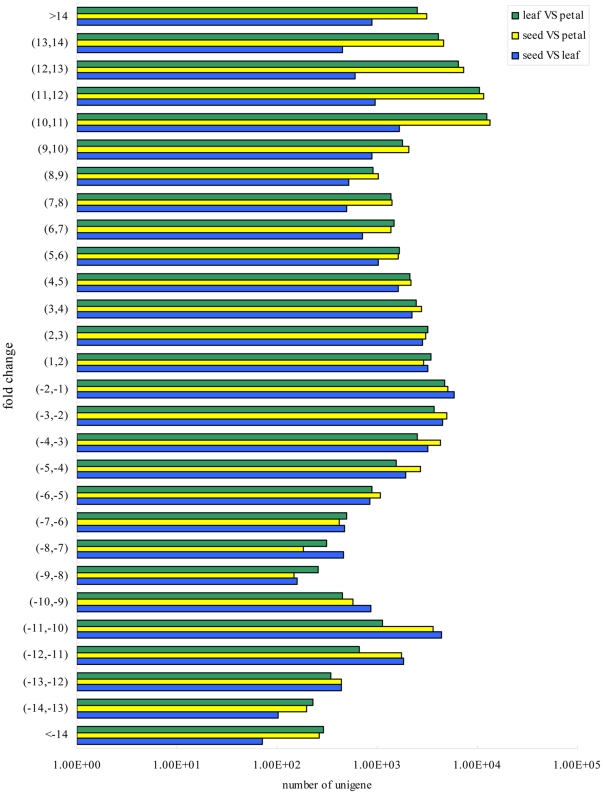
Unigenes that were differentially expressed in the seed, leaf and petal libraries.

To characterize the differentially expressed unigenes in the transcriptome of safflower, we compared the three libraries in pairs of two with the following criteria: absolute value of log_2_ratio >1.0 and P value<0.001). Expression differences between the seed and the leaf libraries revealed that 43,319 unigenes (28.17% of all unigenes) were differentially expressed; 25,157 were up-regulated and 18,162 were down-regulated (figure 5). Among the up-regulated ones, 5,819 unigenes (13.43% of differentially expressed unigenes) were significantly differentially expressed. The most significant change in expression was for unigene144784 which was 16.36 fold more highly expressed in the seed than in the leaf. The second was for unigene58978, annotated as double layer of lipid molecules involved in enclosing cells, which was 16.2 fold more highly expressed in the seed than in the leaf. Compared with in the leaf, in the seed, of the 3,226 down-regulated unigenes, those with log_2_ratios between 1 and 2 formed the largest group (17.76%). The expression of unigene87898, annotated as a chlorophyll-containing plastid, was 21.52 fold lower in the seed than in leaf. When we compared expression differences between the petal and seed libraries, we found that 84,008 unigenes (54.63% of all unigenes) were differentially expressed; 58,351 were up-regulated (69.46% of the differentially expressed sequences) and 25,657 were down-regulated. The log_2_ratio fold changes were from 1 to 17.15 (figure 5). The majority (5,015) of down-regulated genes showed changes in expression between one and two fold, similar to what we found when comparing the seed and leaf libraries. The most significant expression difference was for unigene56205, annotated as a regulator of cellular transcription, which was 16.9 fold lower in the petal than in the seed. Of the up-regulated unigenes, unigene21406, annotated as involved in the catalysis of the transfer of groups such as methyl group, glycosyl group, acyl group and phosphorus-containing, was 21.02 fold more highly expressed in the petal than in the seed. Most unigenes that showed expression changes of only 1 or 2 fold were annotated to be involved in common metabolic processes, such as carbohydrate metabolism; however, unigene5517 (1.15 fold more highly expressed in the petal than in the seed) was annotated as responsible for pigmentation which mostly takes place in the petal. Finally, we found that the expression changes of many of the genes that were related to metabolic pathways were significant between the leaf and petal. We found that 71,994 unigenes (54.63% of all) were significantly differently expressed in the leaf compared to the petal; 54,483 were up-regulated and 17,511 were down-regulated. These differentially expressed unigenes include genes that were predicted to be involved in carbohydrate, amino acid, lipid, and secondary metabolite metabolism. For example, the expression of unigene76334, which was annotated as helicase RecG that functions in DNA recombinase and cell growth [15], was 16.98 fold more highly expressed in the leaf compared with the petal. The expression of unigne135951, annotated as glutathione S-transferase that plays a role in metabolism and stress tolerance, was 18.53 fold lower in the leaf than in the petal.

To investigate the oleosins of seed, we observed the expression levels of oleosins among seed, leaf and petal libraries. Among the unigenes annotated as oleosins mentioned above, unigene24748 and unigene80266 were found to show significant expressions level in seed compared with leaf and petal. We represented the expression level of unigene24748 as 2542.92 RPKM (reads per kilobase of transcript per million reads) in the seed, 13.728 RPKM in the leaf and 2.856 RPKM in the petal. The expression level of unigne80266 was 2658.81 RPKM in the seed, 123 fold higher than in the leaf; this transcript was not found in the petal. Thus, the GO annotation of oleosin for our transcripts is in line with studies that have reported that oleosin is specifically expressed in the seed. In addition, some of the unigenes that were annotated as storage proteins had similar expression characteristics as the candidate oleosins.

The predicted biosynthesis pathways for the unigenes were different in the different tissue libraries and most of the unigenes involved in these pathways were differentially expressed. In particular, we found that the numerous genes predicted to be involved in flavonoid biosynthesis were significantly up or down-regulated in specific tissues. This is consistent with the earlier observations that flavonoids are, for the most part, found in the petal. A number of unigenes, closely related to the chalcone synthase, that were predicted to be involved in the flavonoid biosynthesis pathway were also found to be up-regulated in the petal library. In particular, the expression levels of unigene46610 and unigene53415, annotated as enzymes that catalyze the conversion of cinnamoyl-CoA to pinocembrin chalcone, were greatly changed. Significantly, unigene53415 was expressed only in the petal. Unigenes annotated as related to chalcone isomerase and beta-carotene hydroxylase were up-regulated in the petal. Only unigene75439, annotated as naringenin 3-dioxygenase and predicted to be involved in flavonoid biosynthesis, was down-regulated in this pathway.

### qRT-PCR validation

**Figure 6:**
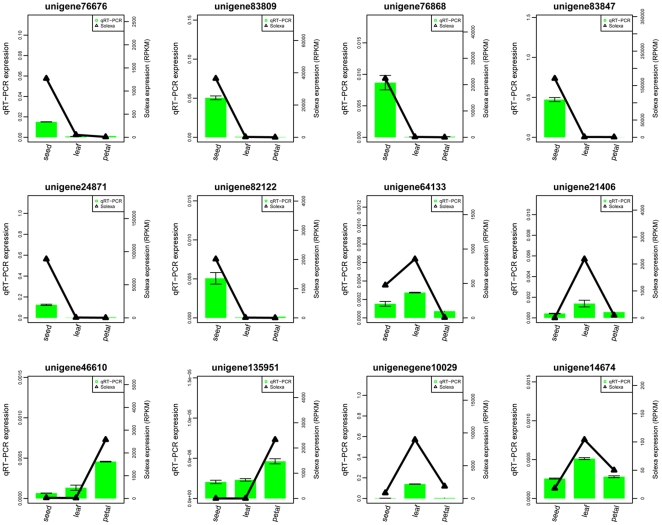
qRT-PCR validation of the selected unigenes.

To confirm the results of the Solexa/Illumina sequencing, thirteen unigenes were selected for quantitative RT-PCR assays. The selected thirteen unigenes showed differential expression patterns related to seed, leaf and petal. We found that qRT-PCR validation of one unigene (unigene5517) was not consistent with sequencing results. Expressions of twelve genes were consistent between the qRT-PCR and the Solexa analyses (figure 6). Five genes annotated to oleosin were validated as being highly expressed in seed; (unigene76676 and unigene83809 responsible for storage of seed lipid, unigene76868 and unigene83847 with hydrophobic core of oil body, unigene24871 identified as a putative oleosin gene). Three tissue-specific genes were also validated: unigene82122 annotated as a putative seed-specific protein; unigene64133 identified as a leaf-specific function; and unigene21406, a high expressed gene related with catalysis in petal. In addition, unigene46610 encoding a protein that was predicted to catalyze the cinnamoyl-CoA to pinocembrin chalcone conversion, and unigene135951 annotated as a glutathione S-transferase was also up-regulated in petal. The differential expressed patterns of unigene10029 and unigene14674 (up-regulated in leaf compared with in petal and seed) were consistent between qRT-PCR and Solexa analyses.

## Discussion

Here we report the results of deep sequencing aimed at obtaining transcript coverage of safflower using the Solexa high-throughput sequencing platform. Previously, Solexa was used to estimate gene expression, for small RNA exploration, and to obtain transcriptome coverage. More recently, large scale of transcriptome analysis based on deep sequencing has been used in gene discovery, the analysis of specific transcripts, and the estimation of overall gene expressions at different development stages and/or in different tissues. In studies of maize, transcriptome analysis has contributed to the annotation of the genomic sequence [16], [17]. Solexa is a cost-effective and efficient DNA sequencing approach that has been used to study grape (*Vitis amurensis*) [10], yellow croaker [18], *Salvia miltiorrhiza* [19], and *Populus euphratica* [*20*], producing data on differentially expressed genes or genes probably associated with potential or novel pathways. Illunima/Solexa estimates gene expression by determining the frequency of EST tags making it one of the most popular tools for gene discovery.

Genome and transcriptome resources for safflower were not yet available. We have analyzed the *de novo* transcriptome of some of the tissues of safflower and obtained a large amount of sequence information. Here we report the results of a single run of Solexa with cDNA amplified from safflower seed, leaf and petal which produced 60,269,546 reads from the seed, 51,702 reads from the leaf and 100,650 reads from the petal. Short reads of 75 bp on average were sufficient to make it possible to annotate more than 153,769 unigenes. From these unigenes we identified 39,190 sequences of more than 300 bp in the seed library and a similar large number in the other two libraries. Our *de novo* analysis identified 43,319 unigenes that were differentially expressed in the seed and leaf, indicating that tissue-specific diversities in seed remain to be further explored. Similar results were observed in the leaf and petal. This result is similar to an earlier study of grape [10].

In the previous study of maize brace root [21], transcriptome dynamics associated with root development were investigated. Millions of transcripts were generated from the stem node tissues and the most differentially expressed tags and the most enriched functional categories were putative protein, metabolism, signal transduction and cellular transport, suggesting that the development of plant tissue is complicatedly regulated requiring the participation of many transcription factors. In the present study, the unigenes annotated as oleosins (e.g. unigene24748, unigene24871, and unigene29297) were highly expressed only in the seed library. It has been reported previously that oleosin is specifically expressed in the seed [22] where it is embedded in the oil body [23] maintaining its morphology [24]. An earlier study also reported that oleosin was most abundantly expressed in the seed [25] of safflower and they were significantly more highly expressed in the seed library with read numbers of more than several thousand. These studies all indicate the importance of the oleosins in the developing and mature oil body where they play a role in stabilizing fatty acid storage in seeds. Oleosins have a hydrophobic central domain and a proline motif at the C-terminal domain of the amino acid sequence [26]. In the present research, our sequence analysis indicated that three of eleven candidate oleosins (unigene80266, unigne83847 and uninge76868) had hydrophobic amino acid sequences, demonstrating that the Illumina/Solexa platform produced sequences that were, for the most part, highly accurate.

A large number of differentially expressed genes involved in flavonoid biosynthesis pathways, such as the genes annotated as participating in anthocyanin synthesis and chalcone synthesis, were also found in the present study. Chalcone synthase is the first enzyme in the flavonoid biosynthesis pathway ([27], [28]. We analyzed the expression pattern of 17 novel transcripts related to chalcone biosynthesis and found that most of them were down-regulated in the leaf compared with the petal. We proposed that a sharp increase of transcripts encoding the chalcone synthase might be required for flavonoid biosynthesis in petal because flavonoid composition in mainly responsible for alterations in fruit color and where it is essential for pollen tube growth and germination [29], [30]. Several important genes related to chalcone biosynthesis that were highly expressed in petal were also identified in the safflower unigene dataset. Therefore, the identified changes in gene expression in safflower that facilitate the synthesis of flavonoid may help in the identification of related genes.

In conclusion, the identification of genes involved in oleosin biosynthesis will contribute to future functional studies in the plant and provide a basis for improving production levels in plants or in microbial hosts by metabolic engineering. In our data set, we identified transcripts that encode all the known enzymes involved in the biosynthesis of the flavonoids and established a gene pool containing flavonoid related unique sequences. Further, we annotated a large number of genes involved in the biosynthesis of secondary metabolites and identified a large number of putative genes that may be involved in secondary metabolism pathways. The data set of assembled safflower unigenes presented here will provide the foundation for other functional and comparative genomic studies.

## Materials and Methods

### Sample culture and sequencing preparation

Safflower seeds (kindly provided by Runkang Traditional Chinese Medicine Institute, Heze, Shandong, China) were cultivated in a climatron with the following conditions: temperature 26°C (day) and 20°C (night); humidity 80%; light for 16 h (intensity of illumination at a constant 30,000 lx) and dark for 8 h. Mature seeds, petals in full bloom and flourish leaves were stored at −80°C for further use. Total RNA was extracted separately from the seeds, petals and leaves using Trizol (Invitrogen, USA) following the manufacturer's protocol.

20 µg of total RNA was prepared for Solexa sequencing. Magnetic beads with polyT oligos attached were used for purifying the mRNA from the total RNA. Then the mRNA was cleaved into small fragments with divalent cations at elevated temperature. The fragments were used to synthesize first-strand cDNA using random hexamer adaptors and reverse transcriptase (Invitrogen, USA). Second-strand cDNA was synthesized with RNase H (Invitrogen, USA) and DNA polymerase I (NEB, USA). Fragments of 300 bp with 200 bp insertions were isolated on separation gels. Read lengths of 75 bp were produced using an Illumina GA IIx following the manufacturer's protocol.

### Sequence annotation

Clean reads were obtained by deleting the empty reads, the adaptor sequences, and the low-quality sequences. The clean reads were assembled into contigs and scaffolds based on pair-end information using SOAPdenovo (http://soap.genomics.org.cn/soapdenovo.html). Functional annotation of the unigenes was performed by running our assembly against the NCBI Nr, COG (http://www.ncbi.nlm.nih.gov/COG) and KEGG (http://www.genome.jp/kegg/) databases using BLAST (E-value<1.0e^−5^). The proteins from NCBI Nr database with the highest sequence similarity to the unigenes were used to assign functional annotations to the genes. The GO (Gene Ontology) annotations for the unigenes were determined by Blast2GO [31]. We then used the WEGO software [32] to analyze the GO functional classification for all the unigenes. The expression level of each unigene was estimated by the frequency of clean reads in the corresponding sample.

### Real-time quantitative RT-PCR (qRT-PCR) analysis

Expression of the selected candidate genes was determined using qRT-PCR. Tissue samples were removed from the freezer and ground in liquid nitrogen. The first-strand cDNA fragment was synthesized from total RNA using a Super RT Kit (BioTeke, China). Gene-specific primers were designed base on the gene sequences using Primer express software. Fifteen primer pairs were designed to amplify 20 target genes. Using the obtained sequences, gene specific primers were designed for each target gene for qRT-PCR (table S3). The qRT-PCR was performed with a Stratagene Mx3000P instrument (Agilent, USA) in a final volume of 25 µl containing 2 µl of cDNA, 12.5 µl 2× SYBR premix Ex taq™ (Takara, Japan), and 10 µM of the forward and reverse primers. The thermal cycling conditions were as follows: 40 cycles at 95°C for 5 s for denaturation and 60°C for 20 s for annealing and extension. The 18 s rRNA gene was used to normalize gene expressions [33], [34]. The relative changes in gene expression levels were calculated using the 2^−ΔΔCt^ method.

## Supporting Information

Table S1 **Unigenes annotated as oleosin by GO.**
(DOC)

Table S2 **Pathway annotation of unignenes from safflower.**
(DOC)

Table S3 **Primer sequences of qRT-PCR.**
(DOC)
