# Supplementary material for: De Novo Transcriptome of Safflower and the Identification of Putative Genes for Oleosin and the Biosynthesis of Flavonoids
Source: PLoS One. 2012 Feb 21;7(2):e30987. doi: 10.1371/journal.pone.0030987 (PMC3283594; doi:10.1371/journal.pone.0030987)
Supplement: Table S1 — Unigenes annotated as oleosin by GO. (DOC) [file pone.0030987.s001.doc]

Tabel S1: Unigenes annotated as oleosin by GO

| Unigene ID | length | NR ID | identity | positive | score | E-value | description |
| --- | --- | --- | --- | --- | --- | --- | --- |
| Unigene24748 | 1232 | gi|15824408|gb|AAL09328.1|AF302806_1 | 0.63 | 0.8 | 462 | 1.00E-129 | steroleosin |
| Unigene24871 | 922 | gi|282895703|gb|ADB03184.1| | 0.61 | 0.73 | 155 | 1.00E-36 | oleosin I |
| Unigene29297 | 1457 | gi|15824408|gb|AAL09328.1|AF302806_1 | 0.52 | 0.81 | 89 | 3.00E-16 | steroleosin |
| Unigene42440 | 887 | gi|21311553|gb|AAM46777.1|AF466102_1 | 0.5 | 0.65 | 127 | 2.00E-28 | oleosin |
| Unigene76676 | 415 | gi|1709459|sp|P29530.2|OLEO1_SOYBN | 0.62 | 0.86 | 123 | 1.00E-27 | oleosin isoform A |
| Unigene76868 | 594 | gi|148283921|gb|ABQ57396.1| | 0.55 | 0.71 | 118 | 7.00E-34 | oleosin H-isoform |
| Unigene80266 | 232 | gi|266694|sp|P29529.1|OLEO_HELAN | 0.75 | 0.9 | 117 | 9.00E-26 | oleosin |
| Unigene83809 | 594 | gi|21311775|gb|AAM46847.1|AF498264_1 | 0.73 | 0.85 | 295 | 4.00E-79 | steroleosin-B |
| Unigene83847 | 612 | gi|266694|sp|P29529.1|OLEO_HELAN | 0.65 | 0.76 | 201 | 7.00E-51 | oleosin |
| Unigene120350 | 354 | gi|196122100|gb|ACG69525.1| | 0.36 | 0.46 | 53.1 | 2.00E-06 | steroleosin SLO2-1 |
| Unigene141701 | 244 | gi|18720|emb|CAA43182.1| | 0.65 | 0.85 | 85.5 | 3.00E-16 | 24 kDa oleosin isoform |
