# Supplementary material for: De Novo Transcriptome of Safflower and the Identification of Putative Genes for Oleosin and the Biosynthesis of Flavonoids
Source: PLoS One. 2012 Feb 21;7(2):e30987. doi: 10.1371/journal.pone.0030987 (PMC3283594; doi:10.1371/journal.pone.0030987)
Supplement: Table S2 — Pathway annotation of unignenes from safflower. (DOC) [file pone.0030987.s002.doc]

Table S2: Pathway annotation of unignenes from safflower

| **Pathway term** | | | **pathway ID** | **unigene number** |
| --- | --- | --- | --- | --- |
| Cellular Processes | Behavior | Circadian rhythm - fly | PATH:ko04711 | 51 |
|  |  | Circadian rhythm - mammal | PATH:ko04710 | 10 |
|  |  | Circadian rhythm - plant | PATH:ko04712 | 317 |
|  | Cell Communication | Adherens junction | PATH:ko04520 | 202 |
|  |  | Focal adhesion | PATH:ko04510 | 219 |
|  |  | Gap junction | PATH:ko04540 | 185 |
|  |  | Tight junction | PATH:ko04530 | 275 |
|  | Cell Growth and Death | Apoptosis | PATH:ko04210 | 286 |
|  |  | Cell cycle | PATH:ko04110 | 547 |
|  |  | Cell cycle - yeast | PATH:ko04111 | 496 |
|  |  | Meiosis - yeast | PATH:ko04113 | 322 |
|  |  | p53 signaling pathway | PATH:ko04115 | 182 |
|  | Cell Motility | Regulation of actin cytoskeleton | PATH:ko04810 | 355 |
|  | Circulatory System | Cardiac muscle contraction | PATH:ko04260 | 116 |
|  |  | Vascular smooth muscle contraction | PATH:ko04270 | 231 |
|  | Development | Axon guidance | PATH:ko04360 | 149 |
|  |  | Dorso-ventral axis formation | PATH:ko04320 | 76 |
|  | Endocrine System | Adipocytokine signaling pathway | PATH:ko04920 | 89 |
|  |  | GnRH signaling pathway | PATH:ko04912 | 347 |
|  |  | Insulin signaling pathway | PATH:ko04910 | 528 |
|  |  | Melanogenesis | PATH:ko04916 | 277 |
|  |  | PPAR signaling pathway | PATH:ko03320 | 197 |
|  |  | Progesterone-mediated oocyte maturation | PATH:ko04914 | 277 |
|  |  | Renin - angiotensin system | PATH:ko04614 | 61 |
|  | Immune System | Antigen processing and presentation | PATH:ko04612 | 267 |
|  |  | B cell receptor signaling pathway | PATH:ko04662 | 148 |
|  |  | Chemokine signaling pathway | PATH:ko04062 | 147 |
|  |  | Fc epsilon RI signaling pathway | PATH:ko04664 | 104 |
|  |  | Fc gamma R-mediated phagocytosis | PATH:ko04666 | 497 |
|  |  | Leukocyte transendothelial migration | PATH:ko04670 | 56 |
|  |  | NOD-like receptor signaling pathway | PATH:ko04621 | 158 |
|  |  | Natural killer cell mediated cytotoxicity | PATH:ko04650 | 121 |
|  |  | RIG-I-like receptor signaling pathway | PATH:ko04622 | 77 |
|  |  | T cell receptor signaling pathway | PATH:ko04660 | 126 |
|  |  | Toll-like receptor signaling pathway | PATH:ko04620 | 319 |
|  | Nervous System | Long-term depression | PATH:ko04730 | 145 |
|  |  | Long-term potentiation | PATH:ko04720 | 298 |
|  |  | Neurotrophin signaling pathway | PATH:ko04722 | 509 |
|  | Sensory System | Olfactory transduction | PATH:ko04740 | 127 |
|  |  | Taste transduction | PATH:ko04742 | 19 |
|  | Transport and Catabolism | Endocytosis | PATH:ko04144 | 742 |
|  |  | Lysosome | PATH:ko04142 | 350 |
|  |  | Regulation of autophagy | PATH:ko04140 | 92 |
| Environmental Information Processing | Membrane Transport | ABC transporters | PATH:ko02010 | 268 |
|  |  | Bacterial secretion system | PATH:ko03070 | 55 |
|  |  | Protein export | PATH:ko03060 | 85 |
|  | Signal Transduction | Calcium signaling pathway | PATH:ko04020 | 220 |
|  |  | ErbB signaling pathway | PATH:ko04012 | 143 |
|  |  | Hedgehog signaling pathway | PATH:ko04340 | 67 |
|  |  | Jak-STAT signaling pathway | PATH:ko04630 | 50 |
|  |  | MAPK signaling pathway | PATH:ko04010 | 257 |
|  |  | MAPK signaling pathway - fly | PATH:ko04013 | 76 |
|  |  | MAPK signaling pathway - yeast | PATH:ko04011 | 44 |
|  |  | Notch signaling pathway | PATH:ko04330 | 84 |
|  |  | Phosphatidylinositol signaling system | PATH:ko04070 | 331 |
|  |  | TGF-beta signaling pathway | PATH:ko04350 | 214 |
|  |  | Two-component system | PATH:ko02020 | 104 |
|  |  | VEGF signaling pathway | PATH:ko04370 | 137 |
|  |  | Wnt signaling pathway | PATH:ko04310 | 351 |
|  |  | mTOR signaling pathway | PATH:ko04150 | 194 |
|  | Signaling Molecules and Interaction | ECM-receptor interaction | PATH:ko04512 | 10 |
|  |  | Neuroactive ligand-receptor interaction | PATH:ko04080 | 62 |
| Genetic Information Processing | Folding, Sorting and Degradation | Proteasome | PATH:ko03050 | 395 |
|  |  | RNA degradation | PATH:ko03018 | 571 |
|  |  | SNARE interactions in vesicular transport | PATH:ko04130 | 150 |
|  |  | Ubiquitin mediated proteolysis | PATH:ko04120 | 785 |
|  | Replication and Repair | Base excision repair | PATH:ko03410 | 205 |
|  |  | DNA replication | PATH:ko03030 | 180 |
|  |  | Homologous recombination | PATH:ko03440 | 155 |
|  |  | Mismatch repair | PATH:ko03430 | 175 |
|  |  | Non-homologous end-joining | PATH:ko03450 | 35 |
|  |  | Nucleotide excision repair | PATH:ko03420 | 312 |
|  | Transcription | Basal transcription factors | PATH:ko03022 | 310 |
|  |  | RNA polymerase | PATH:ko03020 | 723 |
|  |  | Spliceosome | PATH:ko03040 | 2225 |
|  | Translation | Aminoacyl-tRNA biosynthesis | PATH:ko00970 | 418 |
|  |  | Ribosome | PATH:ko03010 | 1577 |
| Metabolism | Amino Acid Metabolism | Alanine, aspartate and glutamate metabolism | PATH:ko00250 | 378 |
|  |  | Arginine and proline metabolism | PATH:ko00330 | 332 |
|  |  | Cysteine and methionine metabolism | PATH:ko00270 | 594 |
|  |  | Glycine, serine and threonine metabolism | PATH:ko00260 | 262 |
|  |  | Histidine metabolism | PATH:ko00340 | 138 |
|  |  | Lysine biosynthesis | PATH:ko00300 | 68 |
|  |  | Lysine degradation | PATH:ko00310 | 229 |
|  |  | Phenylalanine metabolism | PATH:ko00360 | 211 |
|  |  | Phenylalanine, tyrosine and tryptophan biosynthesis | PATH:ko00400 | 180 |
|  |  | Tryptophan metabolism | PATH:ko00380 | 325 |
|  |  | Tyrosine metabolism | PATH:ko00350 | 276 |
|  |  | Valine, leucine and isoleucine biosynthesis | PATH:ko00290 | 266 |
|  |  | Valine, leucine and isoleucine degradation | PATH:ko00280 | 359 |
|  | Biosynthesis of Polyketides and Nonribosomal Peptides | Biosynthesis of ansamycins | PATH:ko01051 | 27 |
|  |  | Biosynthesis of siderophore group nonribosomal peptides | PATH:ko01053 | 4 |
|  |  | Biosynthesis of vancomycin group antibiotics | PATH:ko01055 | 24 |
|  |  | Polyketide sugar unit biosynthesis | PATH:ko00523 | 28 |
|  | Biosynthesis of Secondary Metabolites | Anthocyanin biosynthesis | PATH:ko00942 | 20 |
|  |  | Betalain biosynthesis | PATH:ko00965 | 16 |
|  |  | Brassinosteroid biosynthesis | PATH:ko00905 | 22 |
|  |  | Caffeine metabolism | PATH:ko00232 | 10 |
|  |  | Carotenoid biosynthesis | PATH:ko00906 | 137 |
|  |  | Diterpenoid biosynthesis | PATH:ko00904 | 74 |
|  |  | Flavone and flavonol biosynthesis | PATH:ko00944 | 63 |
|  |  | Flavonoid biosynthesis | PATH:ko00941 | 138 |
|  |  | Glucosinolate biosynthesis | PATH:ko00966 | 33 |
|  |  | Indole alkaloid biosynthesis | PATH:ko00901 | 29 |
|  |  | Isoquinoline alkaloid biosynthesis | PATH:ko00950 | 90 |
|  |  | Limonene and pinene degradation | PATH:ko00903 | 464 |
|  |  | Monoterpenoid biosynthesis | PATH:ko00902 | 36 |
|  |  | Novobiocin biosynthesis | PATH:ko00401 | 43 |
|  |  | Phenylpropanoid biosynthesis | PATH:ko00940 | 461 |
|  |  | Stilbenoid, diarylheptanoid and gingerol biosynthesis | PATH:ko00945 | 349 |
|  |  | Streptomycin biosynthesis | PATH:ko00521 | 131 |
|  |  | Terpenoid backbone biosynthesis | PATH:ko00900 | 171 |
|  |  | Tetracycline biosynthesis | PATH:ko00253 | 22 |
|  |  | Tropane, piperidine and pyridine alkaloid biosynthesis | PATH:ko00960 | 91 |
|  |  | Zeatin biosynthesis | PATH:ko00908 | 32 |
|  | Carbohydrate Metabolism | Amino sugar and nucleotide sugar metabolism | PATH:ko00520 | 430 |
|  |  | Ascorbate and aldarate metabolism | PATH:ko00053 | 194 |
|  |  | Butanoate metabolism | PATH:ko00650 | 356 |
|  |  | C5-Branched dibasic acid metabolism | PATH:ko00660 | 31 |
|  |  | Citrate cycle (TCA cycle) | PATH:ko00020 | 419 |
|  |  | Fructose and mannose metabolism | PATH:ko00051 | 353 |
|  |  | Galactose metabolism | PATH:ko00052 | 300 |
|  |  | Glycolysis / Gluconeogenesis | PATH:ko00010 | 692 |
|  |  | Glyoxylate and dicarboxylate metabolism | PATH:ko00630 | 281 |
|  |  | Inositol phosphate metabolism | PATH:ko00562 | 273 |
|  |  | Pentose and glucuronate interconversions | PATH:ko00040 | 321 |
|  |  | Pentose phosphate pathway | PATH:ko00030 | 290 |
|  |  | Propanoate metabolism | PATH:ko00640 | 311 |
|  |  | Pyruvate metabolism | PATH:ko00620 | 442 |
|  |  | Starch and sucrose metabolism | PATH:ko00500 | 887 |
|  | Energy Metabolism | Carbon fixation in photosynthetic organisms | PATH:ko00710 | 359 |
|  |  | Methane metabolism | PATH:ko00680 | 195 |
|  |  | Nitrogen metabolism | PATH:ko00910 | 252 |
|  |  | Oxidative phosphorylation | PATH:ko00190 | 733 |
|  |  | Photosynthesis | PATH:ko00195 | 133 |
|  |  | Photosynthesis - antenna proteins | PATH:ko00196 | 41 |
|  |  | Reductive carboxylate cycle (CO2 fixation) | PATH:ko00720 | 184 |
|  |  | Sulfur metabolism | PATH:ko00920 | 126 |
|  | Glycan Biosynthesis and Metabolism | Glycosaminoglycan degradation | PATH:ko00531 | 31 |
|  |  | Glycosphingolipid biosynthesis - ganglioseries | PATH:ko00604 | 20 |
|  |  | Glycosphingolipid biosynthesis - globoseries | PATH:ko00603 | 25 |
|  |  | Glycosphingolipid biosynthesis - lacto and neolacto series | PATH:ko00601 | 2 |
|  |  | Glycosylphosphatidylinositol(GPI)-anchor biosynthesis | PATH:ko00563 | 60 |
|  |  | Heparan sulfate biosynthesis | PATH:ko00534 | 19 |
|  |  | High-mannose type N-glycan biosynthesis | PATH:ko00513 | 27 |
|  |  | Lipopolysaccharide biosynthesis | PATH:ko00540 | 18 |
|  |  | N-Glycan biosynthesis | PATH:ko00510 | 206 |
|  |  | O-Glycan biosynthesis | PATH:ko00512 | 38 |
|  |  | O-Mannosyl glycan biosynthesis | PATH:ko00514 | 11 |
|  |  | Other glycan degradation | PATH:ko00511 | 95 |
|  |  | Peptidoglycan biosynthesis | PATH:ko00550 | 2 |
|  | Lipid Metabolism | Androgen and estrogen metabolism | PATH:ko00150 | 53 |
|  |  | Arachidonic acid metabolism | PATH:ko00590 | 46 |
|  |  | Biosynthesis of unsaturated fatty acids | PATH:ko01040 | 318 |
|  |  | C21-Steroid hormone metabolism | PATH:ko00140 | 17 |
|  |  | Ether lipid metabolism | PATH:ko00565 | 217 |
|  |  | Fatty acid biosynthesis | PATH:ko00061 | 272 |
|  |  | Fatty acid elongation in mitochondria | PATH:ko00062 | 19 |
|  |  | Fatty acid metabolism | PATH:ko00071 | 345 |
|  |  | Glycerolipid metabolism | PATH:ko00561 | 230 |
|  |  | Glycerophospholipid metabolism | PATH:ko00564 | 392 |
|  |  | Linoleic acid metabolism | PATH:ko00591 | 152 |
|  |  | Primary bile acid biosynthesis | PATH:ko00120 | 13 |
|  |  | Sphingolipid metabolism | PATH:ko00600 | 147 |
|  |  | Steroid biosynthesis | PATH:ko00100 | 171 |
|  |  | Synthesis and degradation of ketone bodies | PATH:ko00072 | 53 |
|  |  | alpha-Linolenic acid metabolism | PATH:ko00592 | 259 |
|  | Metabolism of Cofactors and Vitamins | Biotin metabolism | PATH:ko00780 | 26 |
|  |  | Folate biosynthesis | PATH:ko00790 | 40 |
|  |  | Lipoic acid metabolism | PATH:ko00785 | 19 |
|  |  | Nicotinate and nicotinamide metabolism | PATH:ko00760 | 61 |
|  |  | One carbon pool by folate | PATH:ko00670 | 123 |
|  |  | Pantothenate and CoA biosynthesis | PATH:ko00770 | 136 |
|  |  | Porphyrin and chlorophyll metabolism | PATH:ko00860 | 153 |
|  |  | Retinol metabolism | PATH:ko00830 | 141 |
|  |  | Riboflavin metabolism | PATH:ko00740 | 55 |
|  |  | Thiamine metabolism | PATH:ko00730 | 50 |
|  |  | Ubiquinone and other terpenoid-quinone biosynthesis | PATH:ko00130 | 149 |
|  |  | Vitamin B6 metabolism | PATH:ko00750 | 31 |
|  | Metabolism of Other Amino Acids | Cyanoamino acid metabolism | PATH:ko00460 | 225 |
|  |  | D-Glutamine and D-glutamate metabolism | PATH:ko00471 | 8 |
|  |  | Glutathione metabolism | PATH:ko00480 | 289 |
|  |  | Phosphonate and phosphinate metabolism | PATH:ko00440 | 22 |
|  |  | Selenoamino acid metabolism | PATH:ko00450 | 202 |
|  |  | Taurine and hypotaurine metabolism | PATH:ko00430 | 44 |
|  |  | beta-Alanine metabolism | PATH:ko00410 | 225 |
|  | Nucleotide Metabolism | Purine metabolism | PATH:ko00230 | 1225 |
|  |  | Pyrimidine metabolism | PATH:ko00240 | 1026 |
|  | Xenobiotics Biodegradation and Metabolism | 1,1,1-Trichloro-2,2-bis(4-chlorophenyl)ethane (DDT) degradation | PATH:ko00351 | 23 |
|  |  | 1,2-Dichloroethane degradation | PATH:ko00631 | 51 |
|  |  | 1,4-Dichlorobenzene degradation | PATH:ko00627 | 21 |
|  |  | 1- and 2-Methylnaphthalene degradation | PATH:ko00624 | 128 |
|  |  | 2,4-Dichlorobenzoate degradation | PATH:ko00623 | 2 |
|  |  | 3-Chloroacrylic acid degradation | PATH:ko00641 | 113 |
|  |  | Atrazine degradation | PATH:ko00791 | 19 |
|  |  | Benzoate degradation via CoA ligation | PATH:ko00632 | 142 |
|  |  | Benzoate degradation via hydroxylation | PATH:ko00362 | 41 |
|  |  | Bisphenol A degradation | PATH:ko00363 | 69 |
|  |  | Caprolactam degradation | PATH:ko00930 | 100 |
|  |  | Drug metabolism - cytochrome P450 | PATH:ko00982 | 196 |
|  |  | Drug metabolism - other enzymes | PATH:ko00983 | 143 |
|  |  | Ethylbenzene degradation | PATH:ko00642 | 40 |
|  |  | Fluorene degradation | PATH:ko00628 | 23 |
|  |  | Fluorobenzoate degradation | PATH:ko00364 | 36 |
|  |  | Geraniol degradation | PATH:ko00281 | 104 |
|  |  | Metabolism of xenobiotics by cytochrome P450 | PATH:ko00980 | 184 |
|  |  | Naphthalene and anthracene degradation | PATH:ko00626 | 371 |
|  |  | Styrene degradation | PATH:ko00643 | 50 |
|  |  | Tetrachloroethene degradation | PATH:ko00625 | 42 |
|  |  | gamma-Hexachlorocyclohexane degradation | PATH:ko00361 | 429 |
