# Supplementary material for: De Novo Transcriptome of Safflower and the Identification of Putative Genes for Oleosin and the Biosynthesis of Flavonoids
Source: PLoS One. 2012 Feb 21;7(2):e30987. doi: 10.1371/journal.pone.0030987 (PMC3283594; doi:10.1371/journal.pone.0030987)
Supplement: Table S3 — Primer sequences of qRT-PCR. (DOC) [file pone.0030987.s003.doc]

**Table S3: Primer sequences of qRT-PCR**

| **gene name** | **primer sequences** | **Tm (℃)** | **Amplicon length (bp)** |
| --- | --- | --- | --- |
| 18s RNA | GAGAAACGGCTACCACATCCAA | 60 | 102 |
|  | TCGTTTGAGCCCGGTATTGTTA |  |  |
| unigene76676 | ATCTTCAGTCCGGTGGTCGTT | 59 | 124 |
|  | GCACATAATTCACCACCGACG |  |  |
| unigene76868 | TTCATAACATCCGGAGCTTTCG | 60 | 134 |
|  | TGGAATCCTGCAGCGATACAC |  |  |
| unigene82122 | ACTTTTGTGACGACGGTGTGG | 60 | 103 |
|  | CGGTTTTCCTCCCTAGACACCT |  |  |
| unigene83809 | CTGCAAAAGCTGCTCTCGTGA | 60 | 105 |
|  | CCATTTCAGCACCGATCCAA |  |  |
| unigene10029 | TGGTTGGCCCTCGTACTATGAA | 60 | 106 |
|  | ATCACAAACCGCACACACGAC |  |  |
| unigene46610 | CGGAGCAGCTTCTCTTCCTTTT | 60 | 107 |
|  | TATGCAGAAGTGCTCGAACGC |  |  |
| unigene14674 | CTTATCCTTCATGGCCTCCCA | 60 | 103 |
|  | GAGCATTTTGGATTGGCGG |  |  |
| unigene5517 | TTATGGTAAAGATGCCGCCG | 59 | 117 |
|  | AAACAGCAGCCACTTGACCAC |  |  |
| unigene64133 | GCATGTGTGCACCTTTGCTTT | 60 | 102 |
|  | ACGGCACACTGAAGGAAATCC |  |  |
| unigene24871 | ATTGATCGCCGTCTTCATCC | 59 | 101 |
|  | CCGTCACGTACGAGTAGATCCA |  |  |
| unigene135951 | GCATTGAGCTGCTCACTTTTGG | 61 | 113 |
|  | GCTGATGTTGATGGACTGCCA |  |  |
| unigene21406 | TCTAGGAACCACTCCAAGTCCA | 57 | 108 |
|  | AGCTGGCTTTTGTTACCGAGA |  |  |
| unigene83847 | GCCTGAGCTCGTTGTCGTACAT | 60 | 105 |
|  | ACATGCCCATATCGTGGATCC |  |  |
